# Kororamides, Convolutamines, and Indole Derivatives as Possible Tau and Dual-Specificity Kinase Inhibitors for Alzheimer’s Disease: A Computational Study

**DOI:** 10.3390/md16100386

**Published:** 2018-10-16

**Authors:** Laura Llorach-Pares, Alfons Nonell-Canals, Conxita Avila, Melchor Sanchez-Martinez

**Affiliations:** 1 Department of Evolutionary Biology, Ecology and Environmental Sciences, Faculty of Biology and Biodiversity Research Institute (IRBio), Universitat de Barcelona, 08028 Barcelona, Catalonia, Spain; laura@mindthebyte.com; 2 Mind the Byte S.L., 08007 Barcelona, Catalonia, Spain; alfons@mindthebyte.com

**Keywords:** meridianins, kororamide A–B, convolutamine I–J, indole scaffold, computer-aided drug design, Alzheimer’s disease, GSK3β, CK1δ, DYRK1A, CLK1

## Abstract

Alzheimer’s disease (AD) is becoming one of the most disturbing health and socioeconomic problems nowadays, as it is a neurodegenerative pathology with no treatment, which is expected to grow further due to population ageing. Actual treatments for AD produce only a modest amelioration of symptoms, although there is a constant ongoing research of new therapeutic strategies oriented to improve the amelioration of the symptoms, and even to completely cure the disease. A principal feature of AD is the presence of neurofibrillary tangles (NFT) induced by the aberrant phosphorylation of the microtubule-associated protein tau in the brains of affected individuals. Glycogen synthetase kinase-3 beta (GSK3β), casein kinase 1 delta (CK1δ), dual-specificity tyrosine phosphorylation regulated kinase 1A (DYRK1A) and dual-specificity kinase cdc2-like kinase 1 (CLK1) have been identified as the principal proteins involved in this process. Due to this, the inhibition of these kinases has been proposed as a plausible therapeutic strategy to fight AD. In this study, we tested in silico the inhibitory activity of different marine natural compounds, as well as newly-designed molecules from some of them, over the mentioned protein kinases, finding some new possible inhibitors with potential therapeutic application.

## 1. Introduction

Constituting about 2% of all human genes, protein kinases are an important family of enzymes with a critical role in signal transduction pathway by modification of substrate activity. They are also responsible to control different aspects of cell functions by its phosphorylation activity, which plays a critical role in intracellular communication during development, and in the function of the nervous and immune systems [1]. Due to that, kinases are related with many diseases, such as Alzheimer’s Disease (AD) or Amyotrophic Lateral Sclerosis (ALS), among others. AD, the neurodegenerative pathology that is considered to represent the most common type of dementia (60–80% of the total cases), is characterized by memory deterioration and modification of cognitive abilities. Alzheimer’s pathologies are associated with the presence of senile plaques (SP), mainly composed by beta-amyloid (Aβ) peptides, and neurofibrillary tangles (NFT), that are intraneuronal aggregations principally composed of abnormal phosphorylated tau protein. Tau is a soluble microtubule-binding protein and is hyperphosphorylated in AD. Tau phosphorylation is regulated by a balance between tau kinase and phosphatase activities. Anti-phosphorylation strategies (kinase inhibitors) aim to inhibit these processes of aggregation and the formation of NFT [2,3,4]. The abovementioned evidence may suggest that one of the key strategies is to prevent tau phosphorylation and, thus, combat AD, could be the inhibition of the protein kinases involved in the tau phosphorylation pathway [4].

Despite the catalytic subunits of many protein kinases are highly conserved, there are several differences between them that allow to classify protein kinases into subfamilies: (1) Protein kinases (EC 2.7.10); (2) serine-threonine protein kinases (EC 2.7.11); (3) dual-specificity kinases (those acting on Ser/Thr and Tyr residues) (EC 2.7.12); (4) protein-histidine kinases (EC 2.7.13); (5) protein-arginine kinases (EC 2.7.11.14); and (6) other protein kinases (EC 2.7.99), that can be also divided into sub-subfamilies, such as tau protein kinase (EC 2.7.11.26) and dual-specificity kinase (EC 2.7.12.1). The main relevant protein kinases involved in tau phosphorylation belong to the sub-subfamilies tau protein kinase and dual-specificity kinases. As tau protein kinases we find glycogen synthetase kinase-3 beta (GSK3β) and casein kinase 1 delta (CK1δ), while within dual-specificity kinases, we find dual-specificity tyrosine phosphorylation regulated kinase 1A (DYRK1A) and cdc2-like kinase 1 (CLK1). Each of them has different roles regarding AD pathology. For GSK3β several authors suggest its link between Aβ and tau pathology, and in AD patients it has been co-localized with NFT. GSK3β is suggested to phosphorylate and hipper-phosphorylate tau, while increasing the production of Aβ and mediating neuronal death. Phosphorylation of tau by GSK3β occurs at 42 sites, where 29 of them are phosphorylated in AD brains. CK1δ is part of the non-proline-directed protein kinase (non-PDPK) group inside the tau kinases and its levels are increased while is co-localized with NFT. CK1δ has an important role on protein aggregation and regulates the microtubule dynamics through tau phosphorylation at 46 sites, 25 of them phosphorylated in AD brains. DYRK1A phosphorylates the amyloid precursor protein (APP) and tau proteins, thus increasing neuronal death and the formation of aggregates. DYRK1A induces tau phosphorylation at serine 202, threonine 212, and serine 404, sites that were found phosphorylated in AD brains. Finally, cdc2-like kinase 1 (CLK1), one of the four isoforms conforming an evolutionary conserved group of dual-specificity kinases, is related with AD by phosphorylating the serine residues in arginine-rich (SR) proteins [2,3,5,6,7,8,9,10,11,12,13,14,15].

The natural-product-inspired design plays an important role in chemical science, as historically natural products (NP) from diverse sources, such as plants or microbes, have been a rich source of compounds [16,17,18]. NP are optimized biologically active metabolites which can be used as a template to design drug-like compounds [16,17,18]. Evaluation of Food and Drug Administration (FDA) approved new molecular entities (NMEs) reveals that NP and their derived compounds represent over one-third of all NMEs [19], a percentage that is even higher regarding the active compounds in the central nervous system (CNS) domain [20]. AD is not an exception, and several drug candidates have been developed from natural sources against the different therapeutic targets identified to date [21,22,23]. In fact, few reasonable selective and potent GSK3β, CK1δ, DYRK1A, and CLK1 inhibitors have been described so far, most of them being marine natural products or derived molecules from them [5,24,25,26,27,28,29,30,31,32,33,34,35,36].

Recently, it has been shown that meridianins, indole alkaloids from the marine tunicate *Aplidium* from the Southern Ocean, could act as inhibitors of these four kinases, with possible inhibitors being derived from them [24,29,34,37]. In addition to that, kororamide A–B, two brominated alkaloids from the bryozoan *Amathia tortuosa* from Australia, showed a phenotypic signature on Parkinson's disease [38]. Their structure resembles that of meridianins and because of that we decided to study whether these compounds could also act as inhibitors of the four mentioned kinases, although, as far as we know, this relation has never been established before. Following with this, and having into account that marine indole alkaloid conform a large group of compounds with diverse biological activities that make them attractive starting points for pharmaceutical development [39,40,41], we have designed in this work several compounds starting from this well-known scaffold as a core element. Further, we modified the structural features observed in meridianins and kororamides, as well as with the presence of halogen substituents (present also in both chemical species), which has been revealed as key player to increase activity over these four kinases [24,37,42,43].

To strengthen our initial assumption, we tested the indole scaffold and halogen substituents’ effect on the inhibition of GSK3β, CK1δ, DYRK1A, and CLK1. To determine the importance of the indole scaffold for the inhibition of the four studied kinases we also screened the MarinLit database [44] to find other possible marine compounds that were similar to meridianin F and kororamide A (which were the best theoretical inhibitors of the four kinases), or at least incorporate the indole scaffold. Thereafter, we analysed their binding behaviour against them. Moreover, and because of the importance of the halogen substituents, we decided to investigate whether the halogen substituents are important with respect to the indole scaffold. To do that, we evaluated the inhibitory behaviour of convolutamine I–J, two halogenated heterocyclic compounds (that do not present an indole scaffold) extracted from the bryozoan *Amathia tortuosa*, and which are structurally and functionally related to kororamide A–B [38].

To sum up, with the general objective to help in the discovery of anti-AD drugs (protein inhibitor/s to reduce or alleviate AD symptoms), the concrete aim of this study is three-fold: (1) to validate if kororamide A–B and convolutamine I–J could act as novel inhibitors of the four studied kinases; (2) to test the indole scaffold importance on the kinases inhibition; and (3) to design new possible inhibitors of the four kinases starting from meridianin and kororamide indole scaffolds. To do so, a computational study targeting the adenosine triphosphate (ATP)-binding site of the aforementioned kinases has been carried out. Computer-aided drug design (CADD) techniques are widely used in (marine natural product) drug discovery, as they constitute an appropriate tool to rational design and developing new drug candidates, reducing the time and costs derived from their identification, characterization, and structure-optimization [45].

## 2. Results and Discussion

### 2.1. New Possible GSK3β, CK1δ, DYRK1A, and CLK1 ATP-Competitive Inhibitors

**Figure 1:**
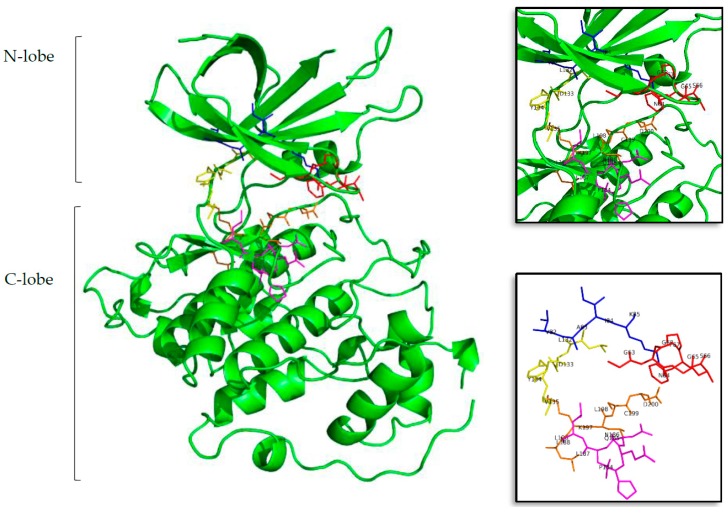
Structure of the tau protein kinase GSK3β (Protein Data Bank ID (PDB) 3PUP). In the first, largest, image the two lobes can be seen in cartoon representation, and in sticks the residues that form the ATP cavity. In the top and bottom zoom images all the amino acid residues involved on the ATP binding site are shown. Residues in red represent the glycine-rich region (GRR), in blue the hydrophobic pocket (HP), in yellow the adenine region (AR), in lilac the sugar pocket (SP), and finally, in orange the phosphate binding pocket (PBP). Letters and numbers correspond to their position in the amino acid sequence and the PDB file numbering.

It is generally accepted that the ATP binding site of protein kinases, despite the fact that their catalytic domains are highly conserved, still remain the most used cavity in (rational) drug design over this family of proteins [46]. Protein kinases have two different lobes, the N-lobe that is mainly formed by β-sheets and the C-lobe formed by a helical structure. Between both lobes can be found the catalytic ATP cavity, which can be divided into five regions: glycine-rich region (GRR), hydrophobic pocket (HP), adenine region (AR), sugar pocket (SP), and the phosphate binding pocket (PBP) [46,47,48]. GRR and HP are located at the N-terminal lobe, while SP and PBP are placed at the C-terminal lobe. AR is in the middle of these regions, providing a link between them (see Figure 1).

All five regions are quite evolutionarily conserved between the kinases, but they are not identical [37]. GRG is a highly conserved region with a GxGxFG motif (Table 1). The same occurs with the HP, as all the four kinases have a VAIK motif, except DYRK1A with a Valine (V) residue instead of an Isoleucine (I). On the contrary, the AR does not seem to have any conserved motif, while SP can be identified by the PxNxL pattern. For the PBP, only the last aspartate residue (D) seems to be conserved along the four kinases.

As explained previously, the kinase ATP binding site is the most exploited cavity as far as inhibition is concerned. Several inhibitors have been reported in the past, some of them being marine natural products, such as meridianins [28,49]. Most of them can bind to all of these regions, with a different binding strength depending on their chemical structure. Interestingly, a common feature seems to be shared between the majority of them: the presence of an indole scaffold [5,25,26,30,31,33,34,35].

### 2.2. Kororamide A–B and Convolutamine I–J as Possible Kinase Inhibitors

**Figure 2:**
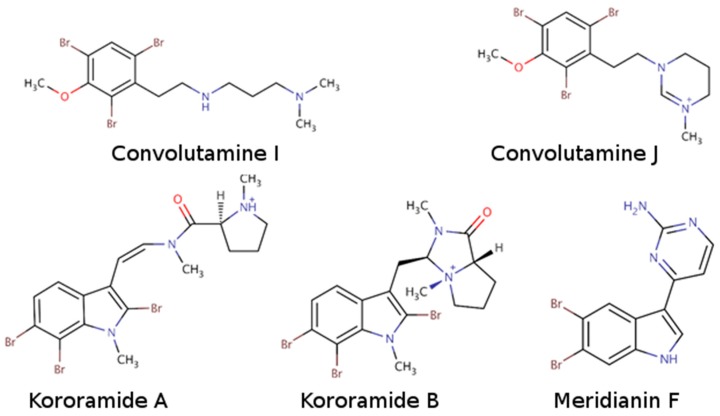
Chemical structures of convolutamine I, convolutamine J, kororamide A, kororamide B, and meridianin F.

Indole alkaloids are marine natural products that show specific biological activities, such as anti-inflammatory and serotonin antagonism [41]. Moreover, the therapeutic importance of this kind of indole scaffolds is well known, as demonstrated by clinical and preclinical studies showing pharmacological activities over neurodegenerative diseases, such as AD [41,50]. Within the group of compounds containing the indole moiety are meridianins, for instance. These molecules constitute a group of indole alkaloids consisting of an indole framework linked to an aminopyrimidine ring with a reported inhibitory activity over GSK3β, CK1δ, DYRK1A, and CLK1 [30,34,37]. Within the list of indole-containing compounds, structurally similar to meridianins, different molecules can be found, among which are kororamides. Kororamide A and B are two tribrominated indole alkaloid compounds from the Southern Ocean bryozoan Amanthia tortuosa. These two marine molecules share a common halogenated indole scaffold with meridianins and, based on their chemical structural similarity, one could assume that kororamides could have an inhibitory activity similar to meridianins. In the same study where kororamide B was identified, three other compounds were also isolated, kororamide A and convolutamine I and J. The last two compounds do not present an indole scaffold, but they are halogenated heterocyclic compounds as other known kinase inhibitors [51,52,53] (Figure 2). To test this hypothesis, docking calculations and Molecular Dynamics (MD) simulations were carried out to evaluate if kororamide A–B and convolutamine I–J could behave as meridianins regarding GSK3β, CK1δ, DYRK1A, and CLK1 binding, thus indicating that they could be potential anti-AD therapeutic agents.

In previous studies the presence of halogen atoms was considered important to achieve a good inhibitory activity over the four studied kinases [24,37]. In order to test whether the presence of a halogenated indole scaffold, or just the presence of aromatic cycle substituted with halogen atoms, enhances a higher binding affinity against GSK3β, CK1δ, DYRK1A, and CLK1, we analyse it by means of docking and MD simulations. Thereafter, we compared the obtained results (Table 2) with the values from kororamide A–B and convolutamine I–J with meridianin F, the most promising compound of the chemical family (meridianin A-F) [37].

**Figure 3:**
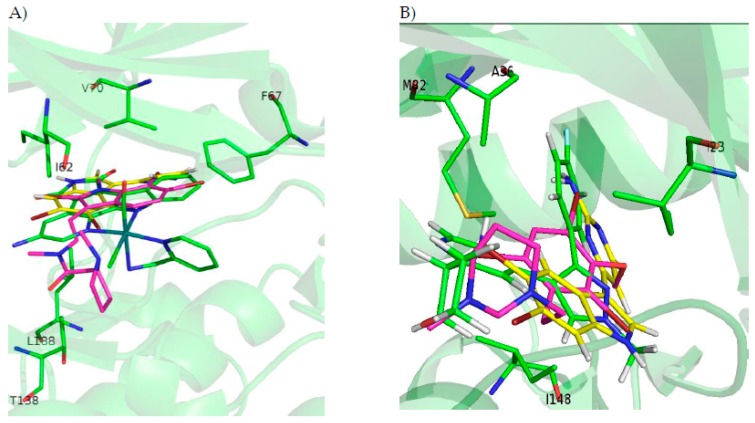

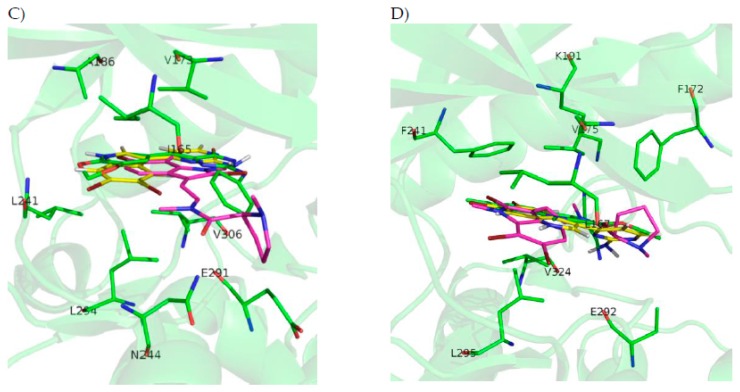
(**A**) ATP cavity site of GSK3β (Protein Data Bank ID (PDB) 3PUP) with meridianin F (yellow), the co-crystallized OS1 inhibitor (green), and the best pose of kororamide A (magenta). (**B**) ATP cavity site of CK1δ (Protein Data Bank ID (PDB) 4KBK) with meridianin F (yellow), the co-crystallized 1QG inhibitor (green), and the best pose of convolutamine J (magenta). (**C**) ATP cavity site of DYRK1A (Protein Data Bank ID (PDB) 4AZE) with meridianin F (yellow), the co-crystallized 3RA inhibitor (green), and the best pose of kororamide A (magenta). (**D**) ATP cavity site of CLK1 (Protein Data Bank ID (PDB) 2VAG) with meridianin F (yellow), the co-crystallized V25 inhibitor (green), and the best pose of kororamide A (magenta). Letters and numbers correspond to their position in the amino acid sequence and the PDB file numbering.

Our results indicate that all the analysed compounds could bind to the ATP binding pocket of each of the mentioned kinases, thus theoretically acting as ATP competitive inhibitors (Figure 3). Binding energies obtained after docking and MD simulations (Table 2) show that convolutamine J and kororamide A tend to have higher energies than convolutamine I and kororamide B. To be more precise, kororamide A shows better energies when bound against GSK3β, DYRK1A, and CLK1, while convolutamine J shows better energies over CK1δ. Comparing the energies obtained between the four tribrominated metabolites found on the bryozoa *Amanthia tortuosa* and meridianin F, we observe that the last one has slightly better energies in all cases after MD. These energies do not allow us to discard any of the compounds as an ATP competitive inhibitor, and we can prioritize kororamide A and convolutamine J over kororamide B, and especially over convolutamine I. Additionally, these results do not allow us to discriminate between which structural features influence most of the binding strength against the four studied kinases: the indole scaffold, the presence of halogen atoms, or the combination of both features.

With the aim of performing a deeper analysis of the inhibitory behaviour of these compounds, an interaction and binding mode analysis, of the best and prioritized compounds per target, was performed. On the ATP catalytic cavity of GSK3β it is observed that key binders I62, F67, and V70, conforming to the GRR or placed nearby, and Y138 and L188 placed at the C-terminal lobe placed near the AR and inside SP, respectively, are involved on the kororamide stabilization. For CK1δ it is observed that convolutamine J is stabilized by interacting with several key binders, like I23, which is placed near the GGR and A36, M82, and I148 placed at HP, AR, and PBP, respectively. Looking at DYRK1A ATP cavity, it is observed that kororamide A, at the N-terminal region, is interacting with I165 and V173, as other known inhibitors like meridianin F or the co-crystal 3RA, both placed near the GRR. In the same way, kororamide A is also stabilized by A185, which is found at the HP. At the C-terminal zone it is also interacting with E291 and the L294 conforming PENIL motif and V306 present at the PBP. Finally, kororamide A is also stabilized by L241 and D244, placed near the AR. Looking at the ATP cavity of CLK1, it can be seen that on the N-terminal domain, L167, F172, and V175 at the GRR, and K191 at the HP, that some of them are known key binders, and are interacting with kororamide A. Moreover, on the C-lobe, kroramide A is interacting with F241 coming from the FELL adenin motif, E292, and L295 (a known key binder), placed at the SP, and V324 found at the PBP.

The binding mode of the best compounds, as well as of the four brominated compounds studied, per target pointed out that they are performing key interactions, most of them previously described in other well-known inhibitors. This fact together with the obtained binding energies, reinforce their capacity to behave as inhibitors for the four analyzed kinases, in a similar way to meridianin F.

### 2.3. Marine Natural Products and Indole Scaffold Validation

With the aim of testing the importance of the indole scaffold as structural key feature on the kinases ATP inhibitors and assuming the well-known Structure Activity Relationship (SAR) principle (i.e., structurally similar compounds will have similar biological activities) a substructure search was performed over the MarinLit database, a dataset that includes revised compounds from marine natural products [44]. In that sense, similar compounds to meridianin F and kororamide A and the indole scaffold were searched over this database. A list of 24 compounds was obtained, 18 compounds when the indole scaffold was used as a seed, and three using meridianin F and kororamide A, respectively. The list could contain more molecules if all the indole-containing compounds were selected. However, we decided that this number is adequate to test if the indole scaffold with several, mostly minor, additions is enough to have a theoretical inhibitory effect over the four kinases, or whether a complex structure like meridianin F or kororamide A is necessary. Docking calculations were performed to analyse the binding behaviour of all of them over GSK3β, CK1δ, DYRK1A, and CLK1 (Table 3).

**Figure A1:**
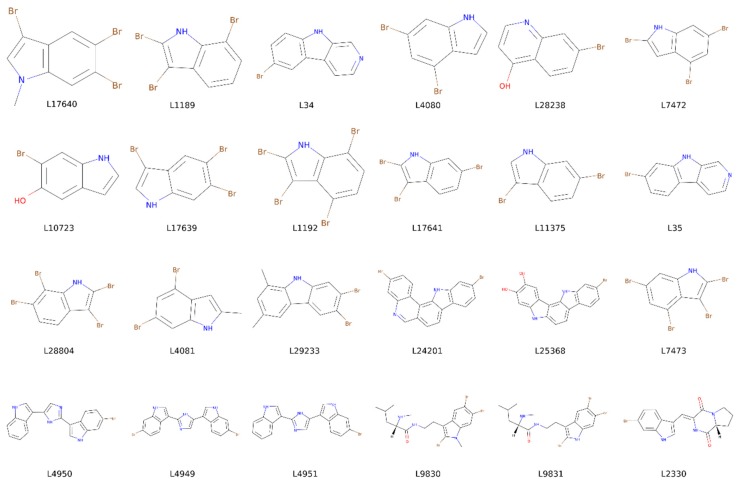
Structures of the 24 compounds found at MarinLit database after a similarity based substructure search using meridianin F, kororamide A, and the indole scaffold as a seed.

All those compounds with energies higher than −9.0 kcal/mol obtained in at least one of the studied targets were considered promising compounds. In fact, after analysing their scaffold, a trend can be seen because all of them have three or more aromatic rings and most of them have two indole scaffolds (Figure A1). Some interesting kinase inhibitors described in recent years corroborate this finding, since they incorporate an indole moiety on their structures [24,25,28,54,55,56].

Moreover, looking at the top ranked compounds, it is easily observed that all of them have a bromine (Br) substituent. Actually, all the 24 compounds have at least one Br atom, a differential signature of marine compounds respect to terrestrial molecules. Many marine organism produce halogenated metabolites unlike terrestrial species [43]. This corroborates the proposed importance of the indole scaffold on the kinases inhibition and seems to point out that the combination of an indole scaffold with halogen substituents could be a good starting point to design new possible inhibitors of the four kinases. This hypothesis is not an isolated fact as marine compounds with this moiety, different to meridianin F and kororamide A, have shown inhibitory effects against some of the studied kinases [24,28].

### 2.4. Indole Derivatives

As mentioned above the SAR hypothesis, widely used in drug discovery, has the premise that structurally-similar molecules have similar biological activities and, thus, similar biological targets. Several known kinase inhibitors possess this moiety and some of them even present a halogenated version of it. In a previous work, we showed that meridianin F, which has a halogenated indole scaffold was the more active member of the family, highlighting the role of this moiety. Now, we have observed that kororamide A and B, given the similarity to meridianin following the SAR principle, could be possible inhibitors of these kinases. This fact is at least partially confirmed (further experiments are needed for a complete validation) because of the in silico obtained binding energies over GSK3β, CK1δ, DYRK1A, and CLK1, reported above. All these facts together, with the observed results in Table 3, made us hypothesize that starting from an halogenated indole moiety and following structural features extracted from meridianin F and kororamide A, we could design indole derivatives that could become kinase inhibitors. Concretely, the indole group was used as a template for the design of a series of seven analogue compounds with different fragments attached to the R3 position of the indole (compounds **1**–**7**; Table 4) and substituted with different combinations of halogen atoms at positions R1 and R2 (a–g combinations; Table 4). Altogether, 49 compounds were designed.

Marine animals have demonstrated to be rich sources of halogenated metabolites and halogenated compounds have a wide range of biological activities [42]. Most halogenated drugs are fluorine (F), followed by chlorine (Cl) and bromine (Br). Contrastingly, for marine-derived molecules, rather than chlorine, the most prevalent halogen found is Br [57]. Halogenated molecules are interesting therapeutic opportunities and it is estimated that one quarter of the total number of final compounds synthesized have an insertion that involves halogens [58]. Halogenated ligands lead to more stable complexes than non-halogenated ligands, and this is important to explain molecular recognition or to planning a screening study [58,59]. Moreover, the capability of halogen atoms to improve oral absorption, lipophilicity, blood brain barrier (BBB) permeability, metabolic and chemical stability, or even potency is well known [58,60]. Therefore, the three mentioned halogen groups at R1 and R2 positions were introduced and evaluated per compound (**1**–**7** + a–g) with the aim of designing the best possible kinase inhibitors (Table 4).

### 2.5. In Silico Binding and Binding Mode Analysis of Indole Derivatives

To analyse the feasibility of the designed compounds as kinase inhibitors by an in silico binding analysis, their binding mode and binding strength against GSK3β, CK1δ, DYRK1A, and CLK1 were analysed. To start with, docking experiments were performed. A total of 441 poses per target were obtained from the 49 compounds of the set. Thereafter, the binding behaviour of all the poses was analysed, showing that the most populated binding region is, as expected, the ATP cavity. With all these results in hand, best poses per target in terms of binding mode and binding energy were selected to perform short MD simulations, for post-processing docking results. For some derivatives none pose was considered for further studies, as the selection of best compounds was carried out considering not only the binding energy but also the binding mode of each molecule, after an interaction analysis study. The poses that did not present good interactions were discarded. Finally, 166 simulations were carried out, corresponding to diverse poses belonging to 45 compounds for GSK3β, 45 for CLK1, 46 in the case of DYRK1A and 30 for CK1δ. After MD simulations, the binding energies of the target-ligand complexes were estimated by molecular mechanics/generalized born surface area (MM/GBSA) calculations. Table 5 summarizes the binding energies of the best indole derivatives, obtained after MD, per compound (**1**–**7**) and target. The rest of the binding energies obtained per derivative and target are reported at Table A1, Table A2, Table A3 and Table A4, respectively.

As a general result, we observe that all the evaluated compounds present better binding interaction energies against CK1δ, DYRK1A and CLK1 than GSK3β, as observed for meridianins [37]. Additionally, as a general trend, compound **1** and **2** always show better energies than the rest of derivatives, highlighting that the fragments introduced in the pyrrole ring (a ketone and an aromatic ring, respectively) of the indole scaffold at R3 position could have beneficial effects to achieve better inhibitory activities over the ATP-binding site of the four studied kinases. Finally, it must be remarked that the designed compounds that do not work against the kinases are different for each one of them, thus opening the door for exploiting these differences in the future to gain selectivity over the four analysed kinases.

#### 2.5.1. GSK3β

As said, the best docked complexes were selected to perform further analysis. For GSK3β 75 poses were chosen and over them MD simulations were performed. From the total studied set, and with the aim of analysing the diverse derivatives, the best a–g combination for each of the **1**–**7** compounds per target was selected. Over the seven best compounds found after MD simulations in terms of binding energy, further analyses were performed, extracting some interesting features. Focusing on the halogen substituents, the best compounds are always those that contain two Br atoms at R1 and R2 position, reflecting the importance of Br substituents observed in previous studies [24,37].

**Figure 4:**
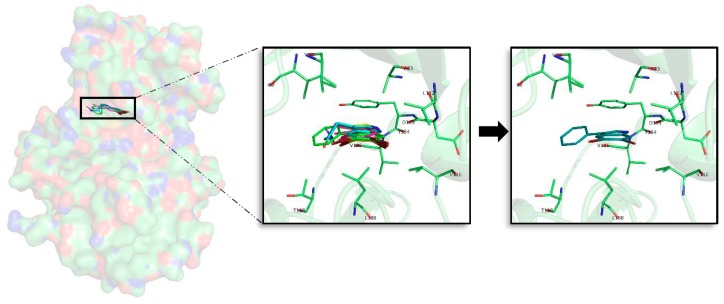
Superposition of the seven best compounds over GSK3β (PDB code: 3PUP) ATP cavity. The active site amino acid residues involved in the binding of the best compounds and the binding position of each of them are enlarged. In the first enlarged panel the 7 top compounds are represented, whereas in the right panel only compound **2a** is shown.

A general pattern regarding the interactions performed by each of the seven best derived compounds at the catalytic ATP binding site was observed. In general, I62, V70, A83, V110, L132, D133, Y134, V135, Y138, and L188 are the most important amino acids for their stabilization over the ATP catalytic pocket (Figure 4). The NH indole group is essential to establish hydrogen bond interactions with the carboxylic acid group (deprotonated under biological conditions) of D133 and/or V135. AR, described by LDYV motif, accommodates the seven best compounds, all of them showing the same binding mode/pose, stabilized by hydrophobic contacts. The indole group is wrapped by N-terminal I62 and V70 residues found near the GRR, together with A83 placed at the HP and C-terminal residues V110 and L188 present at the SP. As the binding mode analysis reveals, all the compounds have the same binding mode, thus binding energy results and MD simulations were used with the aim of identifying some differential features among them. MD analysis reveals that the indole scaffold is maintained wrapped in the same position during all the simulation while the fragments introduced at R3 are more flexible. A binding energy analysis showed that compound **2a** has a slightly better energy than compound **1a**, although both could be considered good plausible options, as the binding energy differences are around 1 kcal/mol, which seems to point out compound **2a** as the best possible inhibitor.

Looking at the literature, our results show that the binding mode displayed by most of the analysed compounds, specially by compound **2a**, correlates with the binding mode of known inhibitors, and also that the residues involved on it are key binders [35,61].

#### 2.5.2. CK1δ

**Figure 5:**
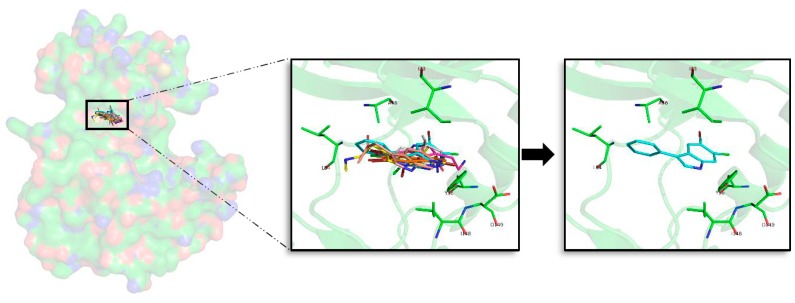
Superposition of the seven best compounds over CK1δ (PDB code: 4KBK) ATP cavity. The active site amino acid residues involved in the binding of the best compounds and the binding position of each of them are enlarged. In the first enlarged panel the seven top compounds are represented, whereas in the right panel only compound **2e** is shown.

For CK1δ, 97 docking poses were subjected to MD simulations. Thereafter, the seven best compounds, in terms of binding energy were selected to be further analysed. Differently to GSK3β, there is not a common binding mode shared by the 7 derived analogues and there is not a specific location of the halogens in the ATP binding site, which can be inferred from the observed binding modes. Although a general pattern could not be observed, there are common features between the studied derivatives than can be highlighted. There is a common behaviour between compounds **1**, **6**, and **7**, and compounds **2**, **4**, and **5** (Figure 5). For the first group the best halogen composition is Br-Br (compound **1a**, compound **6a** and compound **7a**), whereas for the second group, the best halogen composition is **e** (Br-Cl), while for compound **3**, which behaves differently to the rest of the compounds, is **g** (Cl-Br). In all compounds a Br atom is present, which seems to indicate that this presence could be important to increase the binding strength. In general, with few exceptions, the worst binding energies are obtained when there is no Br atom present. This trend is also observed on the rest of kinases (Table A1, Table A2, Table A3 and Table A4). In addition, an accurate analysis of the most important residues involved on the seven compounds binding mode, was performed. This analysis reveals that despite each compound has a different binding pose, there are conserved interactions at the ATP catalytic cavity. According to that, the most important residues on the binding of the seven compounds to CLK1δ are I23, A36, Y56, L84, I148, and D149. All seven derivatives are placed between the HP defined by A36 and the residue I23 that is placed near the GRR, both zones located at the N-terminal region and L84, I149, and D149 placed at the AR and PBP at the C-terminal domain. All the interactions observed between the analogues and the residues are mainly hydrophobic contacts. Binding energies reveal that compound **2e** (Br-Cl) seems to be a slightly better inhibitor than compound **1a**, although both can be considered good options as the energy differences are around 2 kcal/mol.

Different studies have been addressed to find novel and potent CK1δ inhibitors in the last years. Looking at them, it is easy to observe that the interactions made by of all these molecules are aligned, validating it, with the binding mode of our proposed derivates [27,32,33,36].

#### 2.5.3. DYRK1A

**Figure 6:**
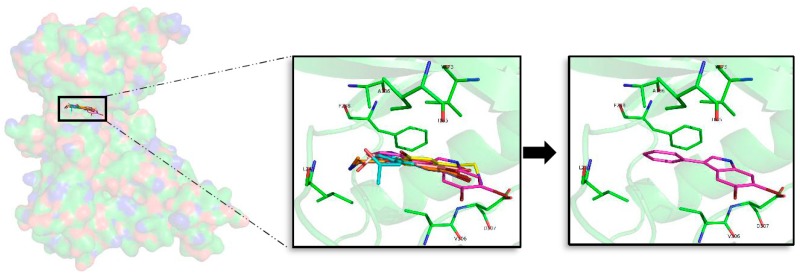
Superposition of the seven best compounds over DYRK1A (PDB code: 4AZE) ATP cavity. The active site amino acid residues involved in the binding of the best compounds and the binding position of each of them are enlarged. In the first enlarged panel the seven top compounds are represented, whereas in the right panel only compound **2a** is shown.

For DYRK1A, 72 docking poses were selected for further analysis. MD simulations were performed over all of them, and thereafter the best compound per target, as for the rest of kinases, was selected. Despite the indole derivatives tested do not shown a shared binding mode as GSK3β, it is more conserved than for CK1δ. All compounds, except compound **3** that is oriented right upside down and moreover shows the worst binding energy, shared the same placement at the ATP catalytic pocket (Figure 6). Analysing the halogen composition of the best compounds it is observed that Br-Br, at R1–R2 positions, is the most common substituent; only compound **1** has a different combination (Br-Cl). As a general conclusion, as with the other three kinases, the presence of at least one Br atom is important to have a good binding affinity.

Looking forward to extract common patterns from the binding modes of the top seven derivatives, it is clear that the seven compounds placed at the catalytic ATP cavity are interacting with I165 and V173, both residues delimitate the GRR, and A186 that is found at the HP, all of them located at the N-terminal region. The important AR formed by a FEML motif also participates on each of the seven bindings, F238 and L241 being the most important residues to stabilize the analysed derivatives. At the C-terminal region, V306 and D307, present at the PBP, are also key binders. Interaction analysis reveals that most of the interactions performed by the derived analogues were hydrophobic contacts. For DYRK1A, after analysing the MD obtained results, it is observed again that compound **2a** is the best derivative in terms of binding energy and binding mode. Interestingly, the observed binding patterns are shared by most of the known inhibitors of this target, which could be found in the literature. Even more, all of them are proposed as ATP-competitive inhibitors like the derivatives we described here [25,26,34].

#### 2.5.4. CLK1

**Figure 7:**
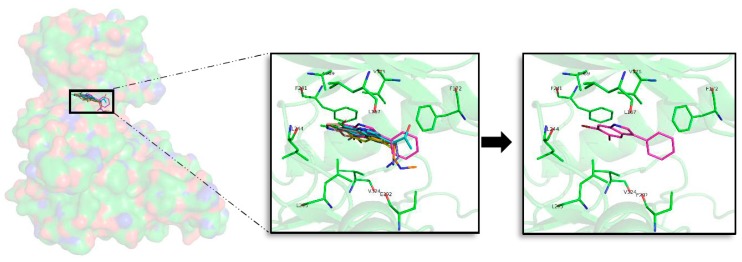
Superposition of the seven best compounds over CLK1 (PDB code: 2VAG) ATP cavity. The active site amino acid residues involved in the binding of the seven best compounds and the binding position of each of them are enlarged. In the first enlarged panel the seven top compounds are represented, whereas in the right panel only compound **2a** is shown.

For CLK1, 87 docking poses were selected for further analysis. All of them were subjected to MD simulations selecting then the best one per target. A first binding mode observation reveals that a common binding mode was found for compounds **1**, **2**, and **6** (Figure 7). These three compounds have the best binding energy, and this could point out the importance of R1, R2 and R3 substituents to gain inhibitory capacity. Compound **3**, despite having a similar binding pose, does not show good energies. The other compounds (**4**, **5**, and **7**) show slightly lower binding energies and a different binding mode, even between them. Focused on the halogen groups, in this target there is not a clear trend, as the seven best compounds show five different halogen substituents (a, c, e, f, and g). Despite this fact, not observed in the rest of studied kinases, a similar trend can be seen. Most of the seven top compounds have a Br atom, except compound **5c**. Moreover, in agreement with the rest of compounds the seven top derivatives are mainly Br or Cl substituents on R1 or R2 position, with the exception of compound **3f**. This seems to suggest that, as for the other targets, all of the analysed halogen substituents combinations could give good inhibitory results, but the presence of a Br is a key factor. In fact, for this target, as seen for the other kinases, the compound **2a** is the best one in terms of binding energy.

A detailed analysis of the displayed binding modes by each compound at the ATP cavity site, reveals interesting shared patterns. On the N-terminal domain L167, F172, and V175 can be found at the GRR, and A189 at the HP acting as key binders. Adenine motif FELL was also revealed important, in particular F241 and L244. On the C-terminal region, residues E292 and L295 at the SP and V324 placed at PBP are the most important amino acids to stabilize the derived compounds over CLK1. As compound **2a**, the other best compounds tend to point their halogen groups between the AR and the HP, fact that facilitate residues as F175 placed at the GRR and E292 or L295 placed at the opposite SP, surround and fixed the indole scaffolds. Interestingly, the binding mode exposed here for the derived analogues in general, and also for the best compound, **2a**, in particular, is validated by other inhibitors reported in the literature [30,31].

The in silico binding studies performed over the four kinases indicate that the derivatives coming from compound **2**, **2a** and **2e**, located at the middle of the ATP binding cavity, seem to be the most plausible ATP competitive inhibitor. However, other derivatives, especially for compound **1** should not be discarded. In general, the presence of the benzene ring at position R3 could have a more positive influence on compounds stabilization at the catalytic site than other substituents. Looking at the literature, several inhibitors described for GSK3β, CK1δ, DYRK1A, and CLK1, as well as other members of the protein kinase family, have aromatic rings in the terminal positions. Moreover, the analysis of the effect of the halogen groups used as substituents at R1 and R2 positions pointed out that its presence can influence the binding strength of the complex (ligand-target). In general, if at least one of the substituents is a Br atom the binding energy is better. An interesting trend found here is that Br seems to be the “best” halogen followed by Cl and F, which, in general, gives worst binding energies. This finding is in line with what is observed in nature, since natural halogenated indole alkaloids contain mostly bromine and chlorine, being the iodinated and fluorinated compounds less abundant [43].

### 2.6. Selectivity

One of the most important challenges on the design of novel kinase inhibitors is the lack of selectivity over the ATP binding site, which is critical in clinical effectiveness of most dugs [46,48]. Most kinase small-molecule inhibitors bind to the ATP catalytic cavity near the AR and wrapped by GRR and HP on the T-lobe and SP and PBP at the C-lobe. The herein performed study does not reveal any significative selectivity over the four kinases for any of the analysed compounds, which could be easily observed looking at the obtained binding modes and energies. However, analysing the residues involved on the binding and the regions occupied by the analogues, some interesting trends that could be exploited in the future can be observed. Interestingly, regarding the binding modes, the best binding energies were obtained on those compounds that are (partially) placed at the PBP. This region, that is very exposed to the solvent and is not usually exploited to gain binding affinity, can be useful to improve the inhibitors selectivity since it contains non-conserved amino acids [46].

Regarding the binding energy results per se, without having into account the binding mode, remarkable significant differences are not observed. The best compound for each target (**2a** and **2e** respectively) comes from the same scaffold, compound **2a** being the best theoretical inhibitor for three of the four targets. If we analyse the binding energies of these top compounds, compounds **2a** and **2e** over DYRK1A and CK1δ, respectively, show a better interaction energy, around 6 kcal/mol of difference, respect to the binding energy of compound **2a** over GSK3β and 3 kcal/mol over CLK1. However, although a slight preference could be inferred from this, the binding of these four compounds to all the four targets is possible with a reasonably good strength. In general, the main differences are observed between the derived compounds **2** (mainly) and **1**, which seems to have better energies than those molecules coming from analogues 3 to 6, and especially with respect to the molecules coming from analogue compound **7** (Table 5). For GSK3β the best compounds coming from derivatives from 2 and 1 (1 kcal/mol of difference between them) are displaying the best interaction energies, followed by those from analogues 4 and 5 (around 4 kcal/mol of difference to compound **2a**), and finally the worst compounds come from analogues 3, 6, and 7 with differences around 13 to 17 kcal/mol respect compound **2a**. In the case of CK1δ, as for GSK3β, the top ranked compounds from analogues 2 and 1 (1.5 kcal/mol of difference between them) have the best binding energies, followed by those from analogues 3–6 with differences around 9 to 11 kcal/mol with respect to compound **2e,** and finally compound **7a** with a difference of around 19 kcal/mol with respect to **2e**. For DYRK1A, the best from compound **2** is the top molecule in terms of interaction energy. Compound **1a** shows a difference of around 5 kcal/mol, whereas compounds **4** and **5** present differences between 6.5 and 7 kcal/mol, respectively, and compounds coming from scaffolds 3, 6, and 7 between 17 and 22.5 kcal/mol. In the case of CLK1, compound **2a** has the better binding energies, followed by those from analogues 1 and 6 (differences around 3 kcal/mol), molecules derived from compounds 4 and 5 (differences around 5 kcal/mol), and finally those from analogues 3 and 7, that show differences around 8.5 to 13 kcal/mol respect to the binding energy

Looking to the 1–7 compounds per target, it can be observed that for compounds **4** and **5** the binding energy differences between the top a–g derivatives range between 2 and 4 kcal/mol between the four kinases. For compounds **1**, **2**, and **7** the differences are higher, ranging between 2.5 and 7 kcal/mol, depending on the compound and target. Finally, for compounds derived from analogues 3 and 6 the differences are even higher, ranging between 8 and 15, and 4 and 12 kcal/mol, respectively. In general, there is not any noticeable selectivity trend derived from the binding energy, although there are some features that could be further exploited. For instance, for DYRK1A and CLK1 an aromatic ring at R3 position is the best choice to gain activity over them, whereas for GSK3β and CK1δ a ketone group at this position could also work, enhancing a way to design selective compounds at least for some of the four kinases.

Exploring the effect of the halogen atoms over the binding strength, as said above, some general trends could be observed but again, its presence does not give any clearly marked or significant selectivity trend between targets. The presence of Br atoms seems to increase the binding strength more than the presence of Cl of F, being in general Cl “better” than F to get good energetic results. However, a possible selectivity feature could be observed due to compound **2e**. Docking energy results are similar for the four kinases, but it only performs good interactions for CK1δ. This is the reason why MD simulation over this compound was only performed in complex with it, while for the other three kinases it was not selected. Compound **2a** gave good docking energies for all four targets but performed good interactions only with GSK3β, DYRK1A and CLK1, so the fact of having a Br-Cl combination at R1-R2 plus an aromatic ring at R3 could be a sign of selectivity over CK1δ, although it should be further explored, as other Cl combinations give good results for the other kinases (Table A1, Table A2, Table A3 and Table A4).

### 2.7. ***2a*** and ***2e*** Unbinding

**Figure 8:**
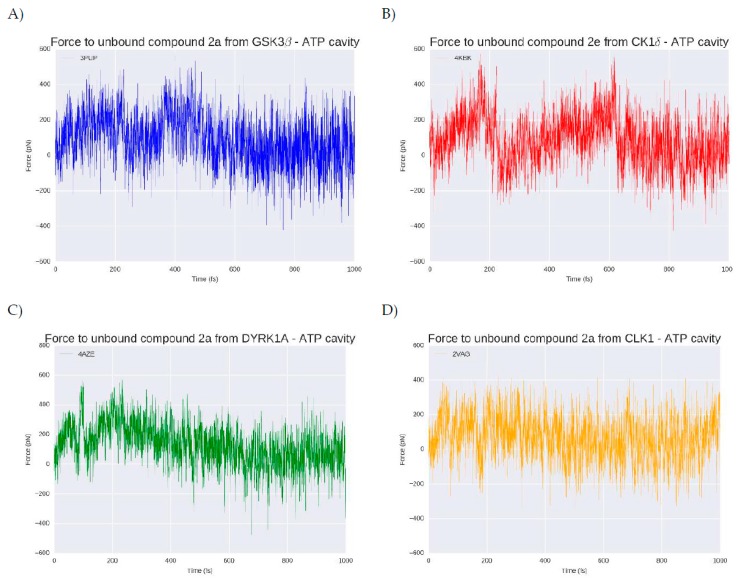
Exerted force in piconewtons (pN) needed to (**A**) remove compound **2a** (blue) from the GSK3β ATP catalytic cavity, (**B**) remove compound **2e** (red) from the CK1δ ATP catalytic cavity, (**C**) remove compound **2a** (green) from the DYRK1A ATP catalytic cavity and remove compound **2a** (orange) from CLK1 ATP catalytic cavity. The x axis represents the computational residence time in femtoseconds (fs).

To reinforce and validate the observed binding trends, as well as to find a differential feature that could help to enhance the selectivity of future derived compounds over the four kinases, steered molecular dynamics (SMD) simulations were performed. Since at the energy and binding mode level there are no significant differences, we intended to see if there was some type of selectivity derived from the protein structure that influences the facility/difficulty of unbinding of the most promising inhibitory compounds **2a** and **2e** per target (Figure 8).

At the beginning of each simulation (t = 0), the compound is in the bound state, placed inside the ATP cavity interacting with the residues previously described. After one nanosecond (ns), at the four kinases, the ligand is completely out of the cavity. In the case of GSK3β (Figure 6A) a force of around 400 pN (piconewtons) is needed to extract compound **2a** from its catalytic cavity. The compound dissociation from the target take place at 200 femtoseconds (fs), moment where the force decrease approach zero pN which means that the compound is out of the cavity. For CK1δ (Figure 7B) that hosts the best compound in terms of binding energy, **2e**, the necessary force to break the ligand-target complex is higher than for the GSK3β complex, with forces that reach up to 600 pN. The ligand unbinding takes place at a similar time than for GSK3β complex, around 200 ps although it takes slightly more time. The dissociation of compound **2a** from DYRK1A (Figure 7C) is done in two phases. A primary rupture force seems to occur before 100 fs, and immediately afterwards it could be observed the highest energy point (around 500 pN), corresponding to the second break. A visual inspection of the SMD confirmed that at this moment, the compound is still inside the ATP pocket. Over 200 fs, the force, after a progressive decrease, arrives to zero pN. This progressive decline correlates with the progressive loose of interactions during the way out of the compound from the catalytic cavity. For the CLK1-compound **2a** complex (Figure 7C) a similar situation is observed. A primary rupture around 100 fs, moment in that, as for DYRK1A, the compound is still placed at the ATP binding site and it is not until later, at 200 fs, when a sudden drop in the energy can be observed, indicating the completely loss of interactions and therefore, the leaving of the cavity. As a general trend, around 200 ps compounds **2a** and **2e** leave the catalytic pocket of the different kinases, requiring a different amount of force that is in line with the observed binding energy. Usually, the better the binding energy, the higher the force needed to break the complex and the longer the residence time. In that sense, the SMD results corroborate what has been seen so far: CK1δ- **2e** and DYRK1A-compound **2a** complexes that have the higher binding energies also seem to have (slightly) longer residence times and require a higher force to take out their respective ligands from their catalytic pockets. There is not any feature that suggests a selectivity trend derived from the unbinding process that could not be extracted from the binding energy results. Compound **2a** is more selective (it binds stronger and requires a higher effort to remove it) for DYRK1A than for GSK3β and CLK1 but could bind to all of them. Compound **2e** seems to be more difficult to unbind that compound **2a**, but this correlates with the higher binding energy it shows after MD.

### 2.8. Pharmacokinetic Properties of Kororamide A–B, Convolutamine I–J, and the Designed Derivatives

Due to the importance of pharmacokinetics (PK) and its impact on drug discovery process, convolutamine I–J, kororamide A–B and the whole set of 49 analogues compounds were analysed, studying their ADME/Tox features. The PK properties of the two best derived compounds **2a** and **2e** are summarized on Table 6 and Table 7. The full set of derivates were also analysed and results can be found at Table A5 (absorption and distribution) and Table A6 (metabolism, excretion and toxicity).

The first PK property analysed was molecular weight, and all compounds show values under 500 Dalton (Da). The higher molecular mass was found for compound **2a** with 351 Da, which is in good agreement with the sizes that a small therapeutic molecule that should cross the BBB should have.

#### 2.8.1. Absorption Properties

Absorption describes the process by which drug candidates move from the point of administration to the blood. LogS descriptor confirmed good solubility in water and good bioavailability for each compound. The derivatives coming from compounds **2** show values that are between −5.1 and −6.1, while for the rest of the derivatives, values are between −3 and −4. Caco-2 permeability revealed medium to high values for all the compounds, except for kororamide A, which was low. The compounds that have a benzene at R3 position as well as the derivatives with F at R1 and R2 positions show moderate permeability and should be optimized in the future. Regarding P-glycoprotein (Pgp) binding, no compound was predicted to act over it. The interaction with Pgp has many pharmacological implications that could result in pharmaceutical advantages or contraindications. For instance, Pgp modulation has been suggested as a mechanism to improve CNS pharmacotherapy [62,63,64,65], but none of the derivatives here seem to have this ability. On the other hand, intestinal absorption values higher than 30% are considered well-absorbed compounds, and for the entire set obtained values are higher than 89%. All of these absorption results suggest good absorption properties for the 49 designed derivatives, plus kororamides and convolutamines.

#### 2.8.2. Distribution Properties

Distribution describes the migration of a compound from the circulation to the extravascular system. LogP values lower than 5 indicate that the compounds have an appropriate hydrophobicity and permeability. In that sense, the derivatives coming from compound **2**, as well as convolutamine J and kororamide A have the highest values (*≈*4) while the rest of compounds are between 2 and 3. Opposite to LogP behaviour, plasma-protein binding (PPB) and steady state volume of distribution (VDss) are not showing as good tendencies for the best derivatives compounds coming from scaffold 1 and 2, and Convolutamine J. Most of the analysed molecules showed medium to high PBP values (except kororamide A and compounds **3b**, **5b**, **6b**, and **7b** with low PPB values) indicating that a high percentage of the administrated compound will be found attached to proteins, affecting its diffusion and its efficiency. As less bound a drug is to plasma proteins, the more efficient it is, as it can traverse cell membranes or diffuse. Regarding VDss, derivatives from scaffolds 3, 6, and 7 and convolutamine I–J plus kororamide A, have high VDss values (>0.45), while for the rest of compounds, its distribution is low to medium, in a close agreement with PBP results. BBB descriptors with results higher than >0.3 reveals good distribution to the brain, as they could pass the blood brain barrier. The highest values are found for convolutamine I, kororamide A, and the derivatives coming from scaffolds 2 and 4, as well as for compounds **5b** and **7b**. However, it should be considered that most of the compounds not predicted to cross BBB have values near the threshold. In addition to BBB, Central nervous system (CNS) permeability was measured. This measure seems to be more precise than BBB, as it is a more direct measurement [66]. Kororamides and convolutamines do not show good permeability values, whereas all the derived compounds showed good results (> −2) allowing us to consider that most of the designed compounds could penetrate the CNS, specially the compounds coming from scaffolds 2 and 4, among which there are the two best candidates **2a** and **2e**.

#### 2.8.3. Metabolism Properties

Cytochrome (CYP) P450 is an important enzyme used to predict drug metabolism. Many drugs could be deactivated or activated by CYP450, as cytochrome P450 enzymes that can be inhibited or induced by drugs, resulting in clinically significant drug-drug interactions that can cause unanticipated adverse reactions or therapeutic failures. Our results revealed that all the analysed compounds, except kororamide B, are likely to be metabolised by CYP450, so their properties should be carefully analysed to design lead compounds from the herein-studied molecules [67,68].

#### 2.8.4. Excretion Properties

Regarding excretion properties, describing the transport of drugs into the urine or bile, good results were obtained. It was found that only kororamide A and B tend to act as a substrate of the organic cation transporter 2 (OCT2 or Solute carrier family 22 member 2, SLC22A2), which means that, in general, and for the two best derivatives **2a** and **2e**, non-clearance problems and adverse interactions with co-administrated OCT2 inhibitors are expected.

#### 2.8.5. Toxicity Properties

During drug development, safety is always the most important issue, including a variety of toxicities and adverse drug effects that should be evaluated in preclinical and clinical trial phases [69]. Between the measured properties, the inhibition of the potassium channels encoded by the human ether-a-go-go gene (hERG) is basic. Our results indicate that none of the compounds seem to be toxic due to hERG. In the same way, none of the designed derivatives is susceptible to be hepatotoxic. However, convolutamine I and J as well as kororamide A tend to be hepatotoxic. Looking at AMES toxicity, which predicts mutagenic and carcinogenic properties, our results revealed that the derivatives from compound 2, as the top derivatives **2a** and **2e**, and kororamide B are predicted to be toxic, while the rest of the set does not. Regarding the maximum recommended tolerated dose (MRTD), the four brominated alkaloids as well as compounds coming from scaffolds 3, 6 and 7 showed low values/doses, which is not the best scenario, whereas the rest of the compounds present good MRTD values.

The well-known Lipinski’s rule of five, formulated in 1997 and that remains in force [70] was also used in combination with the different ADME/Tox properties described above with the aim of evaluate/determine druglikeness of the analysed compounds. To assess how druglike a substance is based on Lipinski’s rules it is accepted that it should have (1) not more than five hydrogen bond donors, (2) ten hydrogen bond acceptors, (3) a molecular mass less than 500 Da, and (4) a LogP not greater than 5. Focusing on the two best compounds (**2a** and **2e**), both have one hydrogen donor and no acceptors. Additionally, as seen in Table 6 and Table 7, the other Lipinski requirements are met. Thus, taking into consideration all the ADMET results described previously, these two compounds can be proposed as good hit candidates, having into account that some properties, such as the possible carcinogenesis and mutagenesis problems should be carefully addressed. In fact, absorption, distribution metabolism, excretion, and toxicity properties should be more or less improved for all the designed compounds, in a further hit to lead (H2L) optimization process. Toxicity should be removed, and compounds interaction with cytochrome P450 carefully analysed and, given the case, eliminated or modulated. Moreover, Caco-2 permeability could be increased, as well as their distribution properties should be improved, lowering the PPB and VDss, to be able to diffuse and penetrate into cells easily.

## 3. Materials and Methods

### 3.1. Computational Virtual Screening

It is well known that there is a correlation between (chemical) structure and (biological) activity, the structure activity relationship (SAR). This SAR is widely exploited in many aspects of the drug discovery pipeline, ranging from compound screening to lead optimization processes, at the experimental and computational levels. Herein, we have performed a 2D virtual screening search over the MarinLit database using its substructure search functionality. Using as an input meridianin F and kororamide A (the two indole compounds that have shown a better binding strength against the four analysed kinases), as well as the indole scaffold alone, a similarity search was performed over MarinLit, obtaining a list of compounds having an indole scaffold in their structure and/or are structurally similar to meridianin F and/or kororamide A. The name and structure of the similar compounds can be found in Figure A1.

### 3.2. Structure Modelling

Convolutamine J, I, and kororamide A and B, were modelled from Dashti et al. [38]. Ligands were prepared to generate energetically-minimized three dimensional (3D) coordinates.

To perform computational work, obtaining good structures to start with is crucial, so prior to any calculation, good computational models should be constructed. The structures of the analysed targets were modelled from 3D crystal structures obtained from the Protein Data Bank (RCSB PDB) [71]. Those structures represented human targets and are the best structures in terms of sequence coverage, of the whole target, in general, and of the binding pocket of each target, in particular. Since all the four kinases biological assembly is in monomeric forms, GSK3β and CK1δ chain B and DYRK1A and CLK1 chain A were respectively selected to perform further studies. To do so, due to the fact that the four studied targets have 3D crystallographic structures, the ATP competitive inhibitor OS1 was co-crystallized with GSK3β (PDB: 3PUP) [35], 1QG was co-crystallized with CK1δ (PDB: 4KBK) [36], the crystal structures of DYRK1A was complexed with 3RA (PDB: 4AZE) [26] and, finally, V25 was co-crystallized with CLK1 (PDB: 2VAG) [31] and used as a template to perform rigid docking calculations using Itzamna (Mind the Byte.SL, Barcelona, Spain) [72].

### 3.3. Docking Calculations

Docking calculations can identify small molecules (ligands) that fit well into the putative binding pocket of a given protein (target) in an optimal way. Without any other specification, generally speaking, docking refers to classical (rigid) docking where only the movement of the ligand is allowed [73]. This kind of calculation allows to elucidate the binding mode, as well as the binding strength of the analysed molecules. Moreover, the molecules could be ranked according to their binding energy. However, this static model is far from real, because proteins are dynamic entities that, to carry out any biological function, need to move. Thus, this movement should be taken into account to obtain good predictions that could be compared with experiments [74,75]. A good option to take the protein movement into account is post-processing docking results by MD simulations [76,77]. MD simulations used to improve docking prediction as they allow observing the induced fit events or, in other words, the conformational adaptation of the target to the ligand, not only the ligand adaptation as happens with rigid docking experiments. Moreover, the stability of the docked complex could be analysed using this pipeline [74,77].

All docking calculations were performed using Itzamna software (Mind the Byte.SL, Barcelona, Spain) [72]. Docking studies were performed between kororamide A–B, convolutamine I–J. and the set of 49 derived compounds against GSK3β, CK1δ, DYRK1A, and CLK1. Two runs were carried out for each calculation to avoid false positives. As the used 3D crystal structures of the kinases were co-crystallized with a ligand, this cavity was employed to dock the analysed compounds for each of the four targets.

### 3.4. Molecular Dynamics Simulations

MD simulations are able to capture the dynamic nature of proteins and bimolecular systems, in general. Herein, short (1 ns) MD simulations were performed using the NAMD program, version 2.11, over the best ligand-target complexes (the top ranked compounds according to the docking binding energies) [78]. The Amber ff99SB-ILDN and the General Amber Force Field (GAFF) set of parameters were employed for modelling receptors and ligands, respectively [79,80], as both forcefields have been extensively tested and used in protein-ligand complexes, giving satisfactory results in several studies [79,80,81,82]. An antechamber was employed to calculate the ligand GAFF parameters and the leap module of Amber Tools to obtain the parameters of the proteins [83,84]. Explicit solvent MD simulations were carried out using a time-step of 2 fs and a TIP3P water model imposing periodic boundary conditions via a cubic box [85]. The distance between the complex and the edge of the box was set to 10 Å. The particle-mesh Ewald method was used to calculate the electrostatic interactions. The temperate and the pressure were kept constant at 300 K and 1 atm, respectively, using Langevin dynamics and a Langevin piston barostat. Bond lengths to hydrogens were constrained with the SHAKE algorithm [86]. Before starting the production simulation, all position restraints were removed, the system was firstly submitted to an energy minimization, following by a solvent equilibration (using harmonic position restraints on the heavy atoms of the protein-ligand complex) and, finally, to a slow heating-up, from 0 to 300 K.

### 3.5. Molecular Mechanics/Generalized Born Surface Area

After performing MD simulations to estimate the ∆G binding free energy of ligand-target complexes, Molecular Mechanics Generalized Born Surface Area continuum solvation (MM/GBSA) reweighting techniques were employed [87]. These techniques outperform docking results because they are employed after MD, thus taking into account the dynamic behaviour of the protein-ligand complexes. However, it should be highlighted that although improve docking binding energy values, are far to be experimentally comparable. Herein, like in other studies, we applied MM/GBSA reweighting techniques over the generated MD trajectories for post-processing docking results [77,88,89]. The MD generated trajectories were employed as input of the MM/GBSA calculations that were realized using the MMPBSA Python algorithm contained within the Amber Tools suite [89].

### 3.6. Steered Molecular Dynamics

Steered molecular dynamics (SMD) is a simulation tool used to explore processes, which cannot usually be achieved by standard MD simulation, such as ligand-protein unbinding or certain protein conformational charges. Here, we have employed it to study ligand unbinding processes. In that sense, in SMD simulations, a time-dependent external force is applied to the ligand, from an internal atom of the protein, to facilitate its unbinding. For a given ligand bound to a target, it allows establishing a theoretical correlation between unbinding forces and residence time and, in turn, its inhibitory capacity; the larger the force and time needed to unbind a ligand from a receptor the higher its binding affinity [90,91,92,93].

SMD simulations were performed using NAMD version 2.11 [78]. Compounds **2a** and **2e** over GSK3β, DYRK1A, CLK1, and CK1δ, respectively, were performed. The last frame obtained from the postprocessing MD simulations was used as an input. A harmonic constraint force constant of 4 kcal/mol/Å with a constant velocity of 0.00002 Å/ns was applied. The time length for each simulation was 1 ns, using a time-step of 2 fs, which was enough to observe the entire ligand unbinding process. The rest of the parameters of the simulations were the same employed for MD simulations. The generated trajectory was finally analysed using visual molecular dynamics (VMD) to extract the exerted force (pN) per simulation frame [94].

### 3.7. Interaction Analysis

To analyse the key residues of the binding pocket involved in the ligand binding, we deeply analysed the obtained binding modes after docking and MD simulations, comparing the obtained results against “known binders” of each of the targets. The “known binders” are important residues for the interaction of reported substrates/inhibitors and were identified at the literature [25,26,27,30,31,32,33,34,35,36,61] as well as in an in-house, recently-constructed database. It was built by crossing ChEMBL and the PDB [62], and it is composed of all PDB structures per UniProt ID with active compounds, plus the residues with which they interact [95,96].

### 3.8. ADME/Tox Properties Prediction

ADME/Tox estimation of the analysed compounds was performed using machine-learning (ML) models. These models were enclosed within the ADMETer software tool (Mind the Byte.SL, Barcelona, Spain) and the pkCSM webserver, respectively [66,97]. Concretely, molecular weight, MRTD, logS, logP, Pgp, caco-2 permeability, BBB penetration, PPB, VDss, CNS penetration, intestinal absorption, AMES toxicity, CYP450 metabolism, hepatotoxicity, hERG binding, and OCT2 binding were predicted. A PgP models were generated by Random Forest against Poongavanam and co-workers (Pgp) and Sedykh et al. datasets [98,99]. The other models and the datasets against the models that were generated are further explained by Llorach-Pares et al. and Pires and co-workers, respectively [37,66].

### 3.9. Graphical Representations

Graphical representations of protein-ligand complexes were prepared using PyMOL version 1.7 and PLIP version 1.3.3 [100,101]. 2D ligand images were prepared using the RDKit [102] Python library and SMD plots using matplotlib [103] and seaborn [104] Python libraries.

## 4. Conclusions

Kororamide A–B and convolutamine I–J can act as tau (GSK3β and CK1δ) and dual-specificity (DYRK1A and CLK1) protein kinase inhibitors. Kororamide A–B are brominated indole alkaloids structurally very similar to meridianins. Only taking this fact into account and following the SAR principle could a kororamides kinase inhibitory effect be hypothesized. Therefore, the in silico binding results we obtained were expected. These results corroborate the idea of that kororamides could be kinase inhibitors with a therapeutic role in AD. Convolutamine I–J, which are not structurally similar to meridianins or kororamides, but are brominated heterocyclic compounds like other known kinase inhibitors, have also shown a plausible inhibitory capacity over GSK3β, CK1δ, DYRK1A, and CLK1. Altogether, the results highlight the role of the indole scaffold and the halogen substituents on these kinase inhibitions, being common features among all the compounds.

However, as happened with several other compounds acting over kinases, their main problem is the selectivity. These compounds seem to be somehow selective for one of the kinases, and it is clear which kinase is the preferred one to bind and which one is undesired but, in general, the obtained energy differences are not enough to consider that these compounds are selective. Moreover, the four brominated alkaloids should be optimized according to their ADMET properties. They have moderated good absorption properties, but caco-2 permeability could be increased, especially for kororamide A, as well as the distribution properties. Additionally, the four compounds show a tendency to have toxicity problems that should be carefully revised, in the same way as compounds interacted with cytochrome P450, although kororamide B does not show this cytochrome interaction.

Through the inclusion of convolutamine into the analysis (as they are brominated but not indole compounds), as well as the exploration of some indole-containing compounds from the MarinLit database, we intended to disentangle whether the indole or the halogen substituents presence is the most important feature to gain activity over the four kinases studied. However, the main conclusion extracted is that, individually, both are equally important, and probably the best way to profit from both features is combining them into halogenated indole scaffolds.

Natural products possess very large therapeutic potential, as reported here and in the related literature. Within natural products, those of unexplored marine origin are of great interest in the discovery of novel chemical structures, since they harbour most of the biodiversity of the world [40,105]. Life started in the oceans and many organisms live only there. Due to this, they should be successfully exploited in the future using sustainability criteria and respecting biodiversity. All this makes computational CADD contributions very relevant, since no biological sample is needed to perform an in silico analysis [106,107,108,109]. Taking all of these facts into account, and profiting from the scaffolds showed by meridianins and kororamides (examples of the importance of halogenated indole scaffolds to gain kinase inhibitory activity), we designed 49 marine natural product derivatives. Concretely, we performed a detailed computational study for the development of specific tau (GSK3β and CK1δ) and dual-specificity (DYRK1A and CLK1) protein kinase inhibitors, starting from marine natural products, meridianin F and kororamide A, until achieving the rational design of indole scaffolds derivates as possible ATP-competitive kinase inhibitors for the treatment of AD. We illustrated how the indole derivate compounds derived from scaffold 2 (an indole with an aromatic ring at R3 position and halogen substituents at R1 and R2), in general, and compounds **2a** and **2e**, in particular, could be proposed as good hit compounds to start an H2L optimization process. Altogether, it could be concluded that kororamides, especially A, convolutamines, especially J, and compounds **2a** and **2e** could be possible ATP-competitive inhibitors against GSK3β, CK1δ, DYRK1A, and CLK1. These results come from in silico experiments and further in vitro and in vivo studies are required. Our results constitute a promising starting point for the development of novel anti-AD drugs.

## Figures and Tables

**Table 1:** Summary of the ATP binding site regions of GSK3β, CK1δ, DYRK1A, and CLK1. Five regions are found inside the ATP cavity and their respective residues are shown in a single letter code, as well as their sequence position that corresponds to each PDB file numbering.

	Glycine-Rich Region	Hydrophobic Pocket	Adenine Region	Sugar Pocket	Phosphate Binding Pocket
**GSK3β**	GNGSFG 63-68	VAIK 82-85	LDYV 132-135	PQNLL 184-188	LKLCD 196-200
**CK1δ**	GSGSFG 16-21	VAIK 35-38	MELL 82-85	PDNFL 131-135	VYIID 145-149
**DYRK1A**	GKGSFG 166-171	VAIK 184-187	FEML 238-241	PENIL 290-294	IKIVD 303-307
**CLK1**	GEGAFG 168-173	VAVK 188-191	FELL 241-244	PENIL 291-295	IKVVD 312-325

**Table 2:** Summary of classical rigid docking and molecular mechanics/generalized born surface area (MM/GBSA) calculations of the two best models selected per meridianin F (F), convolutamine I (I) and J (J), and kororamide A (A) and B (B). To avoid false positives, each docking calculation was performed twice (R0/R1). All energies values are kcal/mol. For each target the first (left) column refers to the results of docking calculations while the second (right) column indicate the binding energy results obtained after MD calculations.

	GSK3β			CK1δ			DYRK1A			CLK1	
	Binding Energy (kcal/mol)	Binding Energy (kcal/mol)		Binding Energy (kcal/mol)	Binding Energy (kcal/mol)		Binding Energy (kcal/mol)	Binding Energy (kcal/mol)		Binding Energy (kcal/mol)	Binding Energy (kcal/mol)
	R0/R1			R0/R1			R0/R1			R0/R1	
**F**	−7.9/−7.9	−35.18	**F**	−7.2/−7.3	−38.55	**F**	−8.0/−7.8	−39.99	**F**	−8.7/−8.7	−37.71
	−7.7/−7.9	−34.73		−7.1/−7.1	−38.93		−7.8/−7.7	−39.91		−8.5/−8.5	−37.61
**I**	−5.6/−5.6	−23.08	**I**	−5.0/−5.0	−3.19	**I**	−5.6/−5.6	−26.52	**I**	−5.8/−5.5	−33.23
	−6.3/−6.3	−18.38		−5.4/−5.4	−11.26		−4.8/−4.8	−11.02		−5.8/−5.8	−31.93
**J**	−6.7/−6.7	−31.58	**J**	−6.2/−6.2	−37.76	**J**	−7.4/−7.4	−31.35	**J**	−6.0/−6.0	−21.47
	−5.9/−5.9	−31.61		−5.8/−5.8	−28.91		−7.0/−7.0	−32.27		−4.6/−4.6	−24.37
**A**	−8.3/−8.3	−34.88	**A**	−8.0/−8.0	−35.48	**A**	−8.2/−8.2	−32.94	**A**	−6.7/−6.7	−37.46
	−8.1/−8.1	−31.02		−7.4/−7.4	−33.94		−6.7/−6.7	−14.61		−2.9/−2.9	−38.93
**B**	−9.1/−9.1	−31.80	**B**	−8.1/−8.1	−28.68	**B**	−7.7/−7.7	−23.83	**B**	−4.4/−4.4	−28.71
	−8.3/−8.3	−32.34		−6.6/−6.6	−35.53		−7.3/−7.3	−24.29		−4.0/−4.0	−22.96

**Table 3:** Summary of classical rigid docking calculations of the marine natural compounds found in the MarinLit database after a substructure similarity search using an indole group, meridianin F, and kororamide A as input molecules. To avoid false positives, each docking calculation was performed twice (R0/R1). All energies values are kcal/mol.

		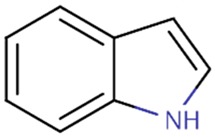					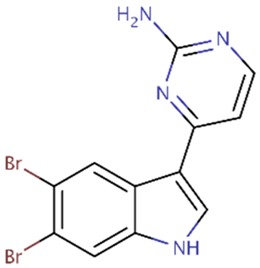		
	**GSK3β**	**CK1δ**	**DYRK1A**	**CLK1**		**GSK3β**	**CK1δ**	**DYRK1A**	**CLK1**
	**Binding Energy**	**Binding Energy**	**Binding Energy**	**Binding Energy**		**Binding Energy**	**Binding Energy**	**Binding Energy**	**Binding Energy**
	**R0/R1**	**R0/R1**	**R0/R1**	**R0/R1**		**R0/R1**	**R0/R1**	**R0/R1**	**R0/R1**
**L17640**	−6.4/−6.4	−7.3/−7.3	−7/−7	−6/−6	**L4950**	−9.1/−9.1	−9.1/−9.1	−9.1/−9.1	−9.1/−9.1
**L1189**	−6.8/−6.8	−7.6/−7.6	−7.2/−7.2	−5.9/−5.9	**L4949**	−8.7/−8.7	−8.7/−8.7	−8.7/−8.7	−8.7/−8.7
**L34**	−7.2/−7.2	−8.1/−8.1	−8.2/−8.2	−6.9/−6.9	**L4951**	−9/−9	−9/−9	−9/−9	−9/−9
**L4080**	−6.1/−6.1	−6.9/−6.9	−6.8/−6.8	−6/−6					
**L28238**	−6.5/−6.5	−7.8/−7.8	−7.3/−7.3	−5.8/−5.8					
**L7472**	−6.3/−6.3	−7.1/−7.1	−6.8/−6.8	−6.2/−6.2					
**L10723**	−6.1/−6.1	−6.7/−6.7	−6.7/−6.7	−5.2/−5.2		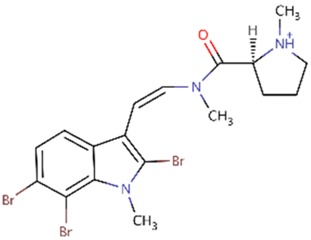			
**L17639**	−6.4/−6.4	−7.1/−7.1	−6.8/−6.8	−5.6/−5.6					
**L1192**	−7.1/−7.1	−7.6/−7.6	−7.6/−7.6	−5.9/−5.9					
**L17641**	−6.8/−6.8	−7/−7	−7/−7	−5.7/−5.7					
**L11375**	−6.2/−6.2	−6.7/−6.7	−6.6/−6.6	−5.4/−5.4					
**L35**	−7.3/−7.3	−8/−8	−8.1/−8.1	−7.1/−7.1		**GSK3β**	**CK1δ**	**DYRK1A**	**CLK1**
**L28804**	−7.1/−7.1	−7.6/−7.6	−7.2/−7.2	−5.8/−5.8		**Binding Energy**	**Binding Energy**	**Binding Energy**	**Binding Energy**
**L4081**	−6.4/−6.4	−7/−7	−6.8/−6.8	−6.4/−6.4					
**L29233**	−8.5/−8.5	−8.5/−8.5	−9.3/−9.3	−8.2/−8.2		**R0/R1**	**R0/R1**	**R0/R1**	**R0/R1**
**L24201**	−10.6/−10.6	−8.8/−8.8	−10.4/−10.4	−9.3/−9.3	**L9830**	−7.4/−7.4	−7.4/−7.4	−7.4/−7.4	−7.4/−7.4
**L25368**	−9.7/−9.7	−8.8/−8.8	−10.3/−10.3	−8.9/−8.9	**L9831**	−7.8/−7.8	−7.8/−7.8	−7.8/−7.8	−7.8/−7.8
**L7473**	−6.9/−6.9	−7.3/−7.3	−7.1/−7.1	−6/−6	**L2330**	−8.7/−8.7	−8.7/−8.7	−8.7/−8.7	−8.7/−8.7

The molecules names (Lxxxx) corresponds to the MarineLit entry code per each compound.

**Table 4:**
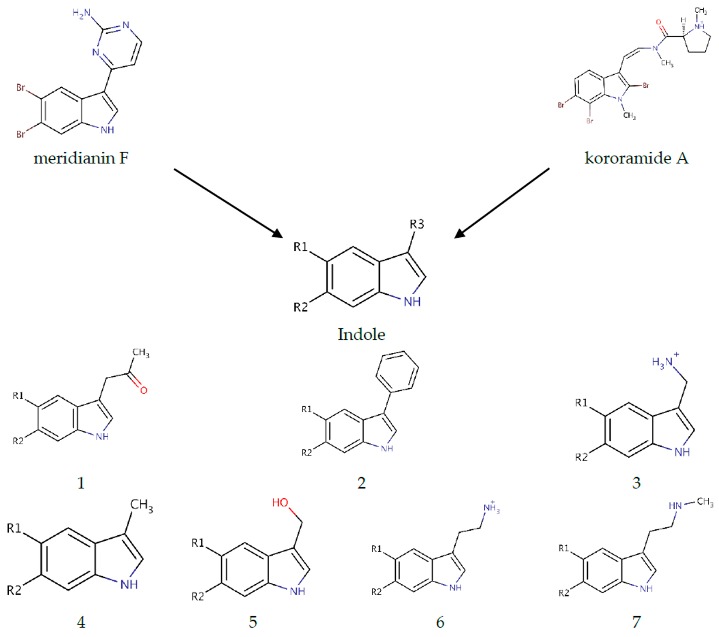
From meridianin F and kororamide A, the indole scaffold was selected to derive new compounds. More precisely, seven indole derivatives were designed (compound **1**–**7**). The R3 position was fulfilled with diverse structural elements mainly inspired on meridianin and kororamide structures. Compound **1** with a ketone group, compound **2** with an aromatic ring, compound **3** with a methylamine, compound **4** with a methyl group, compound **5** with methanol, compound **6** with an ethylamine and compound **7** with an ethyl-methylamine. The R1 and R2 positions were completed with the permutation of Br, Cl and F halogen atoms (a–g) over both positions. At the end 49 indole analogue compounds were designed.

Compounds	R1	R2
**a**	Br	Br
**b**	F	F
**c**	Cl	Cl
**d**	Br	F
**e**	Br	Cl
**f**	F	Br
**g**	Cl	Br

**Table 5:** Summary of molecular mechanics/generalized born surface area (MM/GBSA) calculations of the best derived analogues over the four targets studied. Lowercase letters indicate the halogen substituent group (**a**–**g**), as described in Table 4.

	GSK3β		CK1δ		DYRK1A		CLK1	
	Binding Energy		Binding Energy		Binding Energy		Binding Energy	
Compound **1**	**a**	−30.3141	**a**	−35.4499	**e**	−32.8862	**g**	−30.3541
Compound **2**	**a**	−31.2458	**e**	−37.8982	**a**	−37.8422	**a**	−34.1041
Compound **3**	**a**	−13.8779	**g**	−28.7631	**a**	−15.2733	**f**	−20.4786
Compound **4**	**a**	−27.6481	**e**	−28.6573	**a**	−30.7518	**e**	−28.3695
Compound **5**	**a**	−27.6534	**e**	−28.5831	**a**	−31.2535	**c**	−29.4190
Compound **6**	**a**	−18.5779	**a**	−26.4630	**a**	−18.9387	**g**	−30.7737
Compound **7**	**a**	−18.8955	**a**	−18.4901	**a**	−20.8203	**g**	−25.4765

All energies values are kcal/mol. Maeridianin F results come from our previus publication [37].

**Table 6:** Summary of absorption and distribution properties of the two best compounds **2a** and **2e** found on the four studied kinases. BBB: blood brain barrier, PPB: protein-protein binding, VDss: steady state volume of distribution, CNS: central nervous system.

		Absorption				Distribution				
Compound	Mol Weight	LogS	P-Glycoprotein	Caco-2 Permeability	Intestinal Absorption	LogP	BBB	PPB	VDss	CNS Permeability
Compound **2a**	351	−6.1	inactive	Moderate	90.067	4.1	0.477	High	0.234	−0.894
Compound **2e**	290.1	−5.7	inactive	Moderate	91.036	3.8	0.508	High	0.076	−0.92

**Table 7:** Summary of metabolism, excretion and toxicity properties of the two best compounds **2a** and **2e** found on the four studied kinases. CYP: cytochrome, OCT2: organic cation transporter 2, hERG: human ether-a-go-go gene, MRTD: maximum recommended tolerated dose.

	Metabolism	Excretion	Toxicity			
Compound	CYP450	OCT2 Substrate	hERG	MRTD	AMES Toxicity	Hepatotoxicity
Compound **2a**	Yes	No	<4.0	0.673	Yes	No
Compound **2e**	Yes	No	<4.0	0.641	Yes	No

## Appendix A

**Table A1:** Summary of classical rigid docking calculations of the derived analogues compound set over the GSK3β and Molecular Mechanics/Generalized Born Surface Area (MM/GBSA) calculations of the best posed analogue compounds. Lowercase letters represent the employed halogen group (**a**–**g**).

			GSK3β						
			1	2	3	4	5	6	7
Binding Energy	R0/R1	**a**	−6.9/−6.9	−8.1/−8.1	−5.9/−5.9	−6.5/−6.5	−6.3/−6.3	−6.2/−6.2	−6.6/−6.6
MM/GBSA			−30.3141	−31.2458	−13.8779	−27.6481	−27.6534	−18.5779	−18.8955
Binding Energy	R0/R1	**b**	−6.8/−6.8	−7.9/−7.9	−5.1/−5.1	−6.6/−6.6	−5.6/−5.6	−5.8/−5.8	−5.9/−5.9
MM/GBSA			−22.0902	−23.9910	−7.0321	−17.6371	−19.3959	−9.2248	−20.6117
Binding Energy	R0/R1	**c**	−6.8/−6.8	−8.1/−8.1	−5.8/−5.8	−6.3/−6.3	−6.3/−6.3	−6.2/−6.2	−6.7/−6.7
MM/GBSA			−26.1345	−28.4927	−10.3167	−24.8857	−23.8207	−14.7674	−17.0307
Binding Energy	R0/R1	**d**	−7/−7	−8.1/−8.1	−5.2/−5.2	−6.8/−6.8	−6.5/−6.5	−6.1/−6.1	−6.7/−6.7
MM/GBSA			−26.2805	−29.6158	−12.1225	−22.7717	−23.6754	−14.4347	−13.6539
Binding Energy	R0/R1	**e**	−7/−7	−8.1/−8.1	−6/−6	−6.7/−6.7	−5.8/−5.8	−6.1/−6.1	−6.7/−6.7
MM/GBSA			−27.2898		−19.5564	−25.4497	−26.4593	−17.3314	−18.9577
Binding Energy	R0/R1	**f**	−6/−6	−8.2/−8.2	−5.8/−5.8	−6.3/−6.3	−6.2/−6.2	−6/−6	−6.4/−6.4
MM/GBSA			−26.4517		−6.2475	−23.7315	−19.8909	−13.3104	
Binding Energy	R0/R1	**g**	−6.2/−6.2	−8.2/−8.2	−5.8/−5.8	−6.5/−6.5	−6.2/−6.2	−6.3/−6.3	−5.9/−5.9
MM/GBSA			−28.2864		−14.2501	−24.4026	−24.7124	−16.8986	−20.7272

To avoid false positives, each docking calculation was performed twice (R0/R1). All energies values are kcal/mol.

**Table A2:** Summary of classical rigid docking calculations of the derived analogues compound set over the CK1δ and Molecular Mechanics/Generalized Born Surface Area (MM/GBSA) calculations of the best posed analogue compounds. Lowercase letters represent the employed halogen group (**a**–**g**).

			CK1δ						
			1	2	3	4	5	6	7
Binding Energy	R0/R1	**a**	−5.1/−5.1	−7.7/−7.7	−5.2/−5.2	−5.3/−5.3	−5.6/−5.6	−5.6/−5.6	−5.3/−5.3
MM/GBSA			−35.4499		−3.7149	−26.5327		−26.4630	−18.4901
Binding Energy	R0/R1	**b**	−5.8/−5.8	−7.7/−7.7	−5.5/−5.5	−5.3/−5.3	−5.8/−5.8	−5.6/−5.6	−5.2/−5.2
MM/GBSA			−24.0479	−30.2266		−21.2435		−6.4142	−11.6429
Binding Energy	R0/R1	**c**	−5.6/−5.6	−7.6/−7.6	−4.8/−4.8	−5.7/−5.7	−5.2/−5.2	−5.1/−5.1	−5.6/−5.6
MM/GBSA			−29.9803		−19.4546	−22.8644	−26.4159	−12.0871	
Binding Energy	R0/R1	**d**	−5.9/−5.9	−7.5/−7.5	−4.7/−4.7	−5.5/−5.5	−5.9/−5.9	−5.1/−5.1	−5.5/−5.5
MM/GBSA					−19.8892	−25.5694			
Binding Energy	R0/R1	**e**	−6.1/−6.1	−7.5/−7.5	−5.4/−5.4	−5.3/−5.3	−5.1/−5.1	−5.3/−5.3	−5.3/−5.3
MM/GBSA				−37.8982		−28.6573	−28.5831	−16.2323	
Binding Energy	R0/R1	**f**	−6.2/−6.2	−7.5/−7.5	−5.4/−5.4	−5.2/−5.2	−5.1/−5.1	−5.5/−5.5	−5.4/−5.4
MM/GBSA				−34.6944		−22.6616	−26.3915	−13.6562	−15.4050
Binding Energy	R0/R1	**g**	−6.1/−6.1	−7.3/−7.3	−4.8/−4.8	−5.2/−5.2	−5.1/−5.1	−5.6/−5.6	−5.5/−5.5
MM/GBSA				−33.2393	−28.7631	−26.0731	−28.0238		

To avoid false positives, each docking calculation was performed twice (R0/R1). All energies values are kcal/mol.

**Table A3:** Summary of classical rigid docking calculations of the derived analogues compound set over the DYRK1A and Molecular Mechanics/Generalized Born Surface Area (MM/GBSA) calculations of the best posed analogue compounds. Lowercase letters represent the employed halogen group (**a**–**g**).

			DYRK1A						
			1	2	3	4	5	6	7
Binding Energy	R0/R1	**a**	−6.2/−6.2	−8.6/−8.6	−5.9/−5.9	−6.8/−6.8	−6.7/−6.7	−5.7/−5.7	−6/−6
MM/GBSA				−37.8422	−15.2733	−30.7518	−31.2535	−18.9387	−20.8203
Binding Energy	R0/R1	**b**	−6.5/−6.5	−8.5/−8.5	−7/−7	−7.2/−7.2	−6.6/−6.6	−7/−7	−7.3/−7.3
MM/GBSA			−23.7829	−28.0642	−8.4887	−19.4730	−21.8802	−10.2503	−11.8981
Binding Energy	R0/R1	**c**	−6.3/−6.3	−8.6/−8.6	−5.9/−5.9	−7.2/−7.2	−6.5/−6.5	−6.9/−6.9	−7.3/−7.3
MM/GBSA			−30.2004	−34.1231	−12.1852	−26.5473	−28.3000	−18.7332	−13.2158
Binding Energy	R0/R1	**d**	−5.9/−5.9	−8.6/−8.6	−6.7/−6.7	−6.6/−6.6	−6.3/−6.3	−6.5/−6.5	−6.6/−6.6
MM/GBSA			−30.8597	−36.1125	−10.3667	−26.3748	−26.9602	−17.1653	−16.4510
Binding Energy	R0/R1	**e**	−6.4/−6.4	−8.6/−8.6	−6.5/−6.5	−6.6/−6.6	−6.4/−6.4	−6.6/−6.6	−6.4/−6.4
MM/GBSA			−32.8862		−13.5197	−28.9038	−29.6780		−20.0635
Binding Energy	R0/R1	**f**	−6.2/−6.2	−8.6/−8.6	−5.8/−5.8	−6.8/−6.8	−6.3/−6.3	−6.4/−6.4	−6.9/−6.9
MM/GBSA			−28.9419	−33.0823	−14.5027	−25.4579	−28.4081	−15.8583	−17.0209
Binding Energy	R0/R1	**g**	−6.7/−6.7	−8.7/−8.7	−5.8/−5.8	−6.8/−6.8	−6.7/−6.7	−6.1/−6.1	−6.9/−6.9
MM/GBSA			−30.3186	−35.5805	−13.4928	−27.1510	−29.6010	−18.0146	−17.3973

To avoid false positives, each docking calculation was performed twice (R0/R1). All energies values are kcal/mol.

**Table A4:** Summary of classical rigid docking calculations of the derived analogues compound set over the CLK1 and Molecular Mechanics/Generalized Born Surface Area (MM/GBSA) calculations of the best posed analogue compounds. Lowercase letters represent the employed halogen group (**a**–**g**).

			CLK1						
			1	2	3	4	5	6	7
Binding Energy	R0/R1	**a**	−7.5/−7.5	−8.8/−8.8	−6.4/−6.4	−6.8/−6.8	−6.2/−6.2	−6.4/−6.4	−6.9/−6.9
MM/GBSA			−29.8546	−34.1041	−15.2943	−26.7584	−25.8120	−27.7489	−24.0832
Binding Energy	R0/R1	**b**	−7.1/−7.1	−8.4/−8.4	−7/−7	−5.6/−5.6	−7.5/−7.5	−6.6/−6.6	−7.2/−7.2
MM/GBSA			−25.9089	−27.3711	−13.7919	−26.2326	−22.1539	−16.6984	−20.9149
Binding Energy	R0/R1	**c**	−7.6/−7.6	−9.1/−9.1	−7/−7	−7.3/−7.3	−6.4/−6.4	−6.6/−6.6	−6.4/−6.4
MM/GBSA				−34.0221	−16.2165	−24.6708	−29.4190	−25.8368	−20.9436
Binding Energy	R0/R1	**d**	−7.6/−7.6	−8.4/−8.4	−6.8/−6.8	−7.8/−7.8	−6.3/−6.3	−6.9/−6.9	−7/−7
MM/GBSA			−26.9398	−30.7361		−25.6581	−25.1797	−19.3712	−17.5727
Binding Energy	R0/R1	**e**	−6.8/−6.8	−8.9/−8.9	−6.9/−6.9	−7.5/−7.5	−5.9/−5.9	−7.1/−7.1	−7/−7
MM/GBSA			−30.0891		−16.2097	−28.3695	−28.0697	−27.0478	−17.4985
Binding Energy	R0/R1	**f**	−7.5/−7.5	−8.6/−8.6	−6.7/−6.7	−7.5/−7.5	−6.2/−6.2	−6.4/−6.4	−6.7/−6.7
MM/GBSA				−28.1471	−20.4786	−23.9274	−27.3596	−15.4231	−21.2829
Binding Energy	R0/R1	**g**	−7/−7	−8.9/−8.9	−6/−6	−7.4/−7.4	−6.8/−6.8	−6.4/−6.4	−6.8/−6.8
MM/GBSA			−30.3541	−33.9082	−16.3122	−25.0002		−30.7737	−25.4765

To avoid false positives, each docking calculation was performed twice (R0/R1). All energies values are kcal/mol.

**Table A5:** Summary of absorption and distribution properties of the entire set of 49 derived compounds and the four brominated alkaloids convolutamine I (I) and J (J), kororamide A (A) and B (B).

		Absorption				Distribution				
Compound	Mol Weight	LogS	P-Glycoprotein	Caco-2 permeability	Intestinal Absorption	LogP	BBB	PPB	VDss	CNS Permeability
Compound **1a**	331	−4.8	inactive	High	92.328	2.8	0.298	High	0.231	−1.832
Compound **1b**	209.2	−3.4	inactive	Moderate	94.522	2.6	0.186	Medium	0.131	−1.888
Compound **1c**	242.1	−4.3	inactive	High	92.462	2.9	0.279	High	0.37	−1.832
Compound **1d**	270.1	−4	inactive	High	93.463	2.6	0.152	High	0.249	−1.866
Compound **1e**	286.6	−4.5	inactive	High	92.395	2.8	0.278	High	0.385	−1.832
Compound **1f**	270.1	−4	inactive	High	93.49	2.6	0.152	High	0.256	−1.87
Compound **1g**	286.6	−4.5	inactive	High	92.395	2.8	0.278	High	0.385	−1.832
Compound **2a**	351	−6.1	inactive	Moderate	90.067	4.1	0.477	High	0.234	−0.894
Compound **2b**	229.2	−5.1	inactive	Moderate	92.006	3.4	0.539	High	−0.081	−0.946
Compound **2c**	262.1	−5.9	inactive	Moderate	90.201	4.6	0.482	High	0.197	−0.894
Compound **2d**	306.6	−6.1	inactive	Moderate	90.134	4.4	0.48	High	0.215	−0.894
Compound **2e**	290.1	−5.7	inactive	Moderate	91.036	3.8	0.508	High	0.076	−0.92
Compound **2f**	290.1	−5.7	inactive	Moderate	91.721	3.8	0.708	High	0.051	−1.339
Compound **2g**	306.6	−6.1	inactive	Moderate	90.819	4.4	0.68	High	0.196	−1.313
Compound **3a**	304	−4.7	inactive	High	89.848	2.8	0.227	High	0.95	−1.961
Compound **3b**	182.2	−3.4	inactive	Moderate	91.82	2.7	0.375	Low	0.764	−2.017
Compound **3c**	215.1	−4.2	inactive	High	89.982	2.9	0.23	High	0.919	−1.961
Compound **3d**	243.1	−4	inactive	High	90.899	2.7	0.222	Medium	0.868	−1.999
Compound **3e**	259.5	−4.4	inactive	High	89.915	2.8	0.228	High	0.934	−1.961
Compound **3f**	243.1	−4	inactive	High	90.872	2.7	0.222	Medium	0.845	−1.995
Compound **3g**	259.5	−4.4	inactive	High	89.915	2.8	0.228	High	0.934	−1.961
Compound **4a**	289	−4.9	inactive	High	91.487	3.3	0.351	High	0.432	−1.66
Compound **4b**	167.2	−3.4	inactive	Moderate	93.459	3.2	0.437	Medium	0.248	−1.715
Compound **4c**	200.1	−4.5	inactive	High	91.621	3.6	0.357	High	0.401	−1.66
Compound **4d**	228.1	−4.1	inactive	High	92.538	3.2	0.382	Medium	0.344	−1.697
Compound **4e**	244.5	−4.7	inactive	High	91.554	3.5	0.354	High	0.416	−1.66
Compound **4f**	228.1	−4.1	inactive	High	92.511	3.2	0.382	Medium	0.32	−1.693
Compound **4g**	244.5	−4.7	inactive	High	91.554	3.5	0.354	High	0.416	−1.66
Compound **5a**	305	−4.4	inactive	High	89.763	3	0.284	High	0.253	−1.98
Compound **5b**	183.2	−2.9	inactive	Moderate	91.734	2.6	0.432	Low	0.086	−2.036
Compound **5c**	216.1	−3.6	inactive	High	89.897	2.7	0.287	High	0.223	−1.98
Compound **5d**	244.1	−3.5	inactive	High	90.814	2.7	0.279	Medium	0.169	−2.017
Compound **5e**	260.5	−3.9	inactive	High	89.83	2.9	0.286	High	0.238	−1.98
Compound **5f**	244.1	−3.5	inactive	High	90.786	2.7	0.279	Medium	0.143	−2.014
Compound **5g**	260.5	−3.9	inactive	High	89.83	2.9	0.286	High	0.238	−1.98
Compound **6a**	318	−4.7	inactive	High	90.757	2.7	0.146	High	1.061	−1.917
Compound **6b**	196.2	−3.2	inactive	Moderate	92.728	2.7	0.293	Low	0.867	−1.973
Compound **6c**	229.1	−4.3	inactive	High	90.891	3	0.148	Medium	1.031	−1.917
Compound **6d**	257.1	−3.9	inactive	High	91.78	2.6	0.14	Medium	0.956	−1.951
Compound **6e**	273.6	−4.4	inactive	High	90.824	2.8	0.147	Medium	1.046	−1.917
Compound **6f**	257.1	−3.9	inactive	High	91.808	2.6	0.14	Medium	0.978	−1.954
Compound **6g**	273.6	−4.4	inactive	High	90.824	2.8	0.147	Medium	1.046	−1.917
Compound **7a**	332	−4.9	inactive	High	92.246	3	0.191	High	1.141	−1.487
Compound **7b**	210.2	−3.4	inactive	Moderate	94.218	2.9	0.349	Low	0.994	−1.543
Compound **7c**	243.1	−4.6	inactive	High	92.38	3.3	0.225	Medium	1.11	−1.487
Compound **7d**	271.1	−4.1	inactive	High	93.297	2.9	0.258	Medium	1.084	−1.525
Compound **7e**	287.6	−4.7	inactive	High	92.313	3.2	0.208	High	1.125	−1.487
Compound **7f**	271.1	−4.1	inactive	High	93.27	2.9	0.258	Medium	1.055	−1.521
Compound **7g**	287.6	−4.7	inactive	High	92.313	3.2	0.208	High	1.125	−1.487
J	470	−4.4	inactive	Moderate	90.483	4.4	0.386	High	0.868	−2.215
I	473	−4.3	inactive	Moderate	91.515	3.9	0.193	High	1.474	−2.024
A	534.1	−4.3	inactive	Low	90.979	4.6	0.316	Low	1.112	−2.449
B	535.1	−3.9	inactive	Moderate	100	3.4	0.184	High	0.002	−2.93

BBB: blood brain barrier, PPB: protein-protein binding, VDss: steady state volume of distribution, CNS: central nervous system.

**Table A6:** Summary of metabolism, excretion and toxicity properties of the entire set of 49 derived compounds and the four brominated alkaloids convolutamine I (I) and J (J), kororamide A (A) and B (B).

	Metabolism	Excretion	Toxicity			
Compound	CYP450	OCT2 Substrate	hERG	MRTD	AMES Toxicity	Hepatotoxicity
Compound **1a**	Yes	No	<4.0	0.482	No	No
Compound **1b**	Yes	No	<4.0	0.666	No	No
Compound **1c**	Yes	No	<4.0	0.503	No	No
Compound **1d**	Yes	No	<4.0	0.45	No	No
Compound **1e**	Yes	No	<4.0	0.492	No	No
Compound **1f**	Yes	No	<4.0	0.574	No	No
Compound **1g**	Yes	No	<4.0	0.492	No	No
Compound **2a**	Yes	No	<4.0	0.673	Yes	No
Compound **2b**	Yes	No	<4.0	0.608	Yes	No
Compound **2c**	Yes	No	<4.0	0.671	Yes	No
Compound **2d**	Yes	No	<4.0	0.672	Yes	No
Compound **2e**	Yes	No	<4.0	0.641	Yes	No
Compound **2f**	Yes	No	<4.0	0.585	Yes	No
Compound **2g**	Yes	No	<4.0	0.616	Yes	No
Compound **3a**	Yes	No	<4.0	0.381	No	No
Compound **3b**	Yes	No	<4.0	0.512	No	No
Compound **3c**	Yes	No	<4.0	0.402	No	No
Compound **3d**	Yes	No	<4.0	0.455	No	No
Compound **3e**	Yes	No	<4.0	0.391	No	No
Compound **3f**	Yes	No	<4.0	0.303	No	No
Compound **3g**	Yes	No	<4.0	0.391	No	No
Compound **4a**	Yes	No	<4.0	0.525	No	No
Compound **4b**	Yes	No	<4.0	0.716	No	No
Compound **4c**	Yes	No	<4.0	0.544	No	No
Compound **4d**	Yes	No	<4.0	0.625	No	No
Compound **4e**	Yes	No	<4.0	0.534	No	No
Compound **4f**	Yes	No	<4.0	0.471	No	No
Compound **4g**	Yes	No	<4.0	0.534	No	No
Compound **5a**	Yes	No	<4.0	0.55	No	No
Compound **5b**	Yes	No	<4.0	0.678	No	No
Compound **5c**	Yes	No	<4.0	0.572	No	No
Compound **5d**	Yes	No	<4.0	0.627	No	No
Compound **5e**	Yes	No	<4.0	0.561	No	No
Compound **5f**	Yes	No	<4.0	0.47	No	No
Compound **5g**	Yes	No	<4.0	0.561	No	No
Compound **6a**	Yes	No	<4.0	0.376	No	No
Compound **6b**	Yes	No	<4.0	0.502	No	No
Compound **6c**	Yes	No	<4.0	0.397	No	No
Compound **6d**	Yes	No	<4.0	0.293	No	No
Compound **6e**	Yes	No	<4.0	0.387	No	No
Compound **6f**	Yes	No	<4.0	0.441	No	No
Compound **6g**	Yes	No	<4.0	0.387	No	No
Compound **7a**	Yes	No	<4.0	0.2	No	No
Compound **7b**	Yes	No	<4.0	0.311	No	No
Compound **7c**	Yes	No	<4.0	0.219	No	No
Compound **7d**	Yes	No	<4.0	0.259	No	No
Compound **7e**	Yes	No	<4.0	0.209	No	No
Compound **7f**	Yes	No	<4.0	0.117	No	No
Compound **7g**	Yes	No	<4.0	0.209	No	No
I	Yes	Yes	<4.0	0.029	No	Yes
J	Yes	Yes	<4.0	−0.814	No	Yes
A	Yes	No	<4.0	−0.599	No	Yes
B	No	No	<4.0	0.405	Yes	No

CYP: cytochrome, OCT2: organic cation transporter 2, hERG: human ether-a-go-go gene, MRTD: maximum recommended tolerated dose.
